# Therapeutic effects and potential mechanisms of endoscopic submucosal injection of mesenchymal stem cells on chronic atrophic gastritis

**DOI:** 10.1038/s41598-023-48088-3

**Published:** 2023-11-25

**Authors:** Qianqian Xu, Mingyue Liu, Rui Meng, Qi Zhao, Xiaoxiao Men, Yadi Lan, Hongwei Xu

**Affiliations:** 1 Department of Gastroenterology, Shandong Provincial Hospital, Shandong University, No. 324, Jingwuweiqi Road, Jinan, 250021 Shandong People’s Republic of China; 2 Department of Gastroenterology, Shandong Provincial Hospital Affiliated to Shandong First Medical University, Jinan, 250021 Shandong People’s Republic of China; 3 Department of Gastroenterology, Dezhou People’s Hospital, Dezhou, 253000 Shandong People’s Republic of China; 4 Department of Gastroenterology, Qilu Hospital of Shandong University Dezhou Hospital, Dezhou, 253000 Shandong People’s Republic of China

**Keywords:** Gastritis, Mesenchymal stem cells

## Abstract

Previous studies have demonstrated the rejuvenating and restorative actions of mesenchymal stem cells (MSCs) in multiple diseases, but their role in reversing chronic atrophic gastritis (CAG) is not well understood owing to their low efficiency in homing to the stomach. In this work, we investigated the therapeutic effect of umbilical cord-derived MSCs (UC-MSCs) on CAG by endoscopic submucosal injection and preliminarily explored possible mechanisms in vitro. MSCs and normal saline (NS) were injected into the submucosa of the stomach in randomly grouped CAG rabbits. Therapeutic effects on serum indices and histopathology of the gastric mucosa were analyzed in vivo at 30 and 60 days after MSCs injection. GES-1 cells were co-cultured with MSCs in vitro using a Transwell system and cell viability, proliferation, and migration ability were detected. Additionally, in view of the potential mechanisms, the relative protein expression levels of apoptosis, autophagy and inflammation in vitro were explored by Western Blotting. We found that submucosal injection of MSCs up-regulated serum indices (G-17, PGI and PGI/PGII) and alleviated histopathological damage to the gastric mucosa in CAG rabbits. Co-culture of GES-1 cells with MSCs improved cell viability, proliferation, and migration ability, while suppressing apoptosis. We also observed a reduction in the expression of apoptosis indicators, including Bax and cleaved caspase-3, in GES-1 cells after co-culture with MSCs in vitro. Our findings suggest that submucosal injection of MSCs is a promising approach for reversing CAG, and attenuating apoptosis plays a potential role in this process.

## Introduction

Various factors, including Helicobacter pylori (H. pylori) infection, bile reflux, and autoimmunity, instigate a stepwise progression of lesions in the gastric mucosa, commencing with chronic gastritis, chronic atrophic gastritis (CAG), intestinal metaplasia (IM), dysplasia, and culminating in intestinal-type gastric carcinoma, popularly known as the “Correa's cascade”^1^. CAG, which is manifested as inflammatory infiltration, glandular atrophy, and partial intestinal metaplasia, appears asymptomatic and reversible, making it a focus in the multistep process of carcinogenesis^2^. Data from follow-up indicates that eradication of H. pylori and other etiological treatments can partially reverse atrophy and metaplasia to some extent^3–5^. Nevertheless, certain fundamental questions about atrophy and metaplasia reversal persist. Firstly, identifying the most potent responder cells in the gastric mucosa is crucial. Studies have established the role of isthmic stem cells and chief cells in mucosal reversal^6^. However, their precise mechanisms of action remain elusive. Secondly, the mechanisms underlying atrophy and metaplasia improvement need to be elucidated. Moreover, natural reversal occurs over an extended period, necessitating the discovery of ways to expedite the process.

Mesenchymal stem cells (MSCs) are multipotent stem cells capable of multidirectional differentiation that can be derived from bone marrow (BM-MSCs), umbilical cord (UC-MSCs), or adipose tissue (AD-MSCs)^7^. MSCs secrete a variety of cytokines, chemokines, growth factors, and exosomes that indirectly and remotely aid tissue repair. Importantly, MSCs modulate trophic factors that can mediate anti-apoptotic, antifibrotic, antioxidant, and immunosuppressive effects^8^. Benefits from their easy availability, genetic stability, and low immunogenicity, MSCs have been widely applied in various medical fields such as inflammation, autoimmunity, immune rejection, and degenerative diseases^9^. Recently, the efficacy and potential mechanism of MSCs in the treatment of inflammatory bowel disease^8^, cirrhosis^10^, esophageal stricture^11^, and other gastroenterological diseases have been continuously published. However, owing to the low homing of MSCs, there have been limited studies on their roles and mechanisms in CAG reversal.

In our study, we utilized a rabbit model of CAG and performed endoscopic submucosal injections of MSCs to investigate their therapeutic potential. Through comprehensive analysis of serum and pathological indicators, we demonstrated the efficacy and safety of MSCs in reversing gastric mucosal atrophy. In addition, our work also revealed the underlying mechanisms of MSCs in a cell model induced by N-methyl-N-nitro-N-nitrosoguanidine (MNNG).

## Materials and methods

### Cells

Human umbilical cord blood mesenchymal stem cells (hUC-MSCs) were purchased from Desheng Biological Engineering Co., Ltd. (Shandong, China), cultured in an α-MEM medium containing 10% (v/v) fetal bovine and 100 U/ml penicillin/streptomycin. The normal gastric mucosal epithelial cell line GES-1 were purchased from Procell Life Science & Technology Co., Ltd. (Hubei, China), cultured in an DMEM medium containing 10% (v/v) fetal bovine and 100 U/ml penicillin/streptomycin. Both cell lines were incubated at 37 °C in a humidified 5% CO_2_ incubator.

### Establishment of CAG rabbit model

Healthy adult male rabbits were obtained from Xietong Medicine Bioengineering Co., Ltd. (Jiangsu, China) and were housed under standard conditions with free access to food and water in a clean environment on a 12-h day-night cycle. To simulate the low gastric acidity state induced by H. pylori infection and bile reflux, a rabbit model of CAG was established by chemical stimulation. Rabbits were divided into the model group (n = 20) and the control group (n = 5). The model group was provided with free access to drinking water containing 150 μg/ml MNNG, 0.02% ammonia water, and 20 mmol/L deoxycholic acid, and was fasted twice a week. The control group was given normal water and food. The model was developed over a 12-week period, after which endoscopy and biopsy pathology assessment were conducted. Venous blood samples were also drawn from the ear margins to analyze serum biochemical indices, as outlined in the following procedure. Upon successful model completion, we proceeded with submucosal hUC-MSCs injections. The experimental procedures were approved by the Institutional Animal Care and Committee at Shandong Provincial Hospital, Shandong University, and conducted in compliance with the regulations.

### Endoscopic examination

**Figure 1:**
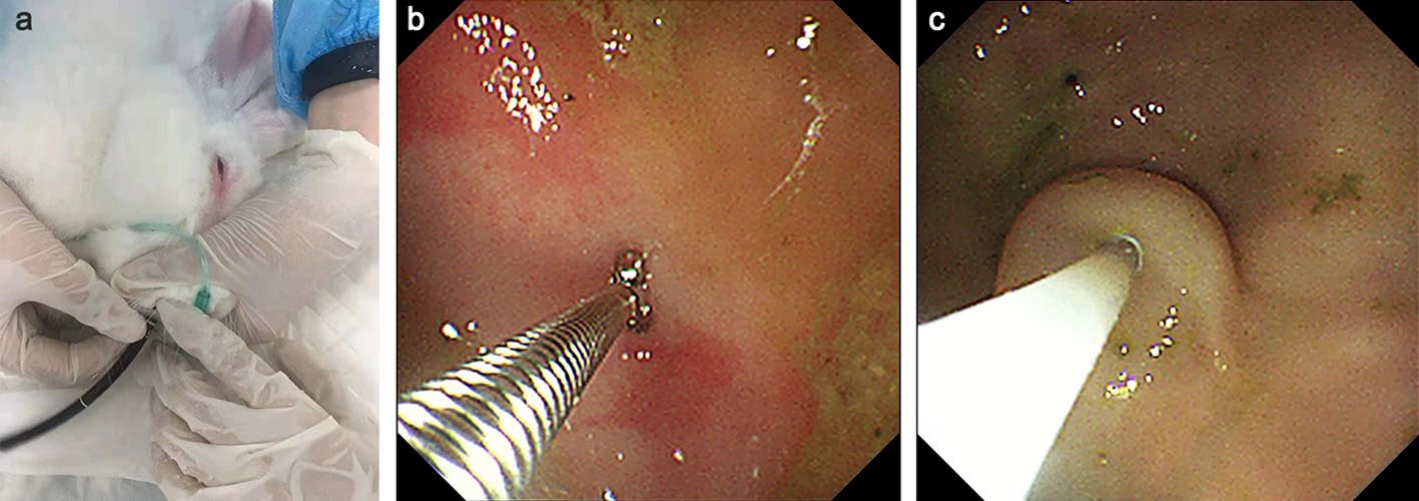
Endoscopic examination and submucosal injection procedure in a rabbit model. (**a**) Rabbit receiving propofol anesthesia and mechanical ventilation during the endoscopic procedure. (**b**) Gastric mucosal biopsy with biopsy forceps. (**c**) Endoscopic image after submucosal injection.

After a 24-h fasting period, the rabbits were anesthetized with propofol (*i.v.* maintenance) and provided with mechanical ventilation during the procedure. The gastroscopic procedure used in rabbits is illustrated in Fig. 1a. Briefly, the rabbit was positioned laterally on the examination table. An electronic transnasal gastroscope (Olympus, GIF-XP260N, Tokyo, Japan) was introduced through the rabbit's oral cavity. To safeguard the endoscope from potential damage by the rabbit's teeth, a five-milliliter syringe cartridge was used as a protective dental cushion. Real-time images from the endoscope's camera were displayed on a monitor (Olympus, CV-170, Tokyo, Japan), aiding in the precise navigation through the esophagus to access the stomach. Gastroscopic images were captured during the examination, and biopsy forceps were utilized through an accessory channel to obtain samples of the gastric mucosa for subsequent histological analysis (Fig. 1b). Upon completion of the examination, the endoscope was carefully withdrawn from the rabbits' digestive tracts. Post-surgery, all animals received 1 ml of preheated saline for postoperative fluid resuscitation. Gastroscopy was performed, and biopsies were taken at the end of the modeling period and at each injection session.

### Submucosal hUC-MSCs injection

To perform submucosal injection, hUC-MSCs were first resuspended in saline at a concentration of 10 × 10^6^/10 ml. The rabbits in the CAG model were divided into the MSCs group (n = 10) and the saline-control group (n = 10). A standardized 10 ml of a different solution was then injected into the submucosal layer at multiple sites of the stomach using a disposable injector (Olympus, NM-201L-0423, Tokyo, Japan) through the accessory channel of the electronic transnasal gastroscope (Fig. 1c). Specifically, we followed the New Sydney System's guidelines for biopsy locations and performed submucosal injections at five specified sites (the antrum at the lesser and greater curvature, the body at the lesser and greater curvature, and the incisura, corresponding to the anatomy of the human stomach). At each site, at least two distinct points were injected. Each injection site received 1 ml, approximately 1 × 10^6^ cells. The injection depth was restricted to the submucosa, and a noticeable elevation in the submucosal layer could be observed after the injection. Each group received two submucosal injections, with one month between treatments. One month after the final injection, gastroscopy and biopsy were once again performed on the rabbits, and peripheral blood samples were collected. At the conclusion of the experiment, all rabbits were humanely euthanized through the process of gas embolization.

### Serum biochemical indices examination

Serum biochemical indices, including gastrin 17 (G-17), pepsinogen I (PG I), and pepsinogen II (PG II), were measured by enzyme-linked immunosorbent assay (ELISA). Kits were purchased from OriGene Wuxi Biotechnology. Inc. (Jiangsu, China).

### Histopathological examination

The resected tissues were fixed in 10% formalin for 2 days, embedded in paraffin, sliced, and stained with hematoxylin and eosin (H&E). Five fields of gastric antrum mucosa were sampled from each section, and the thickness of glands in each field was measured using a micrometer. The average value of the five fields within each section was then calculated (μm). At 100X magnification, the total number of intact glands within the gastric antrum was counted and expressed as "units/mm".

### Co-culture of hUC-MSCs and GES-1

To establish an in vitro model of therapy, a cell–cell co-culture system was implemented using a 24-well plate with a 0.4 μm and 3 μm pore size membranes. The upper surface of the membrane was seeded with 1.0 × 10^5^ degenerative GES-1 cells, while the base of the well was seeded with 3.0 × 10^5^ hUC-MSCs at passage 4–6. The cells were co-cultured in α-MEM for 24 h at 37 °C and 5% CO_2_ in a humidified atmosphere. Prior to co-culture, GES-1 cells were exposed to 40 μmol/L MNNG for 24 h to induce a cell atrophy model.

### Cell migration assay

Following 24-h co-culture in a 24-well plate with a 3 μm pore size membrane, the nutrient solution was removed and the upper layer of the Transwell was gently wiped with a cotton swab. The membrane was then fixed with methanol for 30 min, washed with PBS, and stained with hematoxylin for 10 min. Five fields were randomly selected, and the number of invasive cells was counted under a microscope (Olympus, IX71-F22PH) to obtain the mean value, which was used for statistical analysis.

### Cell proliferation assay

The proliferative capacity of GES-1 cells was assessed using an EdU (5-ethynyl-2′-deoxyuridine) assay, facilitated by the Cell-Light EdU DNA Cell Proliferation Kit (RiboBio, Shanghai, China). Following 24-h co-culture through a 0.4 μm pore size membrane, cells were treated with 100 μl of EdU and incubated for an additional 2 h. Post-treatment, the cells were fixed with 4% paraformaldehyde and stained with Apollo Dye Solution for proliferating cells, while nucleic acid was stained with Hoechst 33342. The cell proliferation rate was determined following the manufacturer's guidelines. Fluorescence images were captured using a fluorescence microscope (Invitrogen, EVOS M7000).

### Western blot

Total protein was extracted from cells using RIPA buffer containing 1/100 phenylmethylsulfonyl fluoride (PMSF; Solarbio, Beijing, China). The lysate was centrifuged at 12,500 g for 10 min at 4 °C and the supernatant was collected for protein quantification using a BCA protein assay kit (Solarbio, Beijing, China). Equal amounts of soluble protein were denatured by heating at 100 °C for 10 min in loading buffer (Solarbio, Beijing, China). The denatured proteins were then separated by sodium dodecyl sulfate–polyacrylamide gel electrophoresis (SDS-PAGE) and transferred onto polyvinylidene fluoride (PVDF) membranes. The membranes were blocked in 5% skimmed milk powder (SMP) in TBST at room temperature for 2 h. Next, the membranes were incubated overnight at 4 °C with specific primary antibodies. On the following day, the blots were washed and incubated with HRP-conjugated secondary antibodies for 1 h at room temperature, followed by another wash. Chemiluminescent signals were visualized using ECL Substrate (Sparkjade, Shandong, China) and detected on a ChemiDocMP System (Biorad, United States) for analysis.

### Experimental design and statistical analysis

Experimental design, analysis and reporting followed the ARRIVE guidelines (https://www.nc3rs.org.uk/arrive-guidelines) where possible. Statistical analyses were conducted using GraphPad Prism 8 and SPSS 25.0. Differences with statistical significance were determined by unpaired Student’s t-test. Chi-square or Fisher’s exact test were applied to compare categorical variables across the groups. The pathological score per sample was computed as the aggregate of all recorded findings. A *P*-value of less than 0.05 was considered statistically significant.

## Results

### Therapeutic effects of MSCs on rabbit CAG

**Figure 2:**
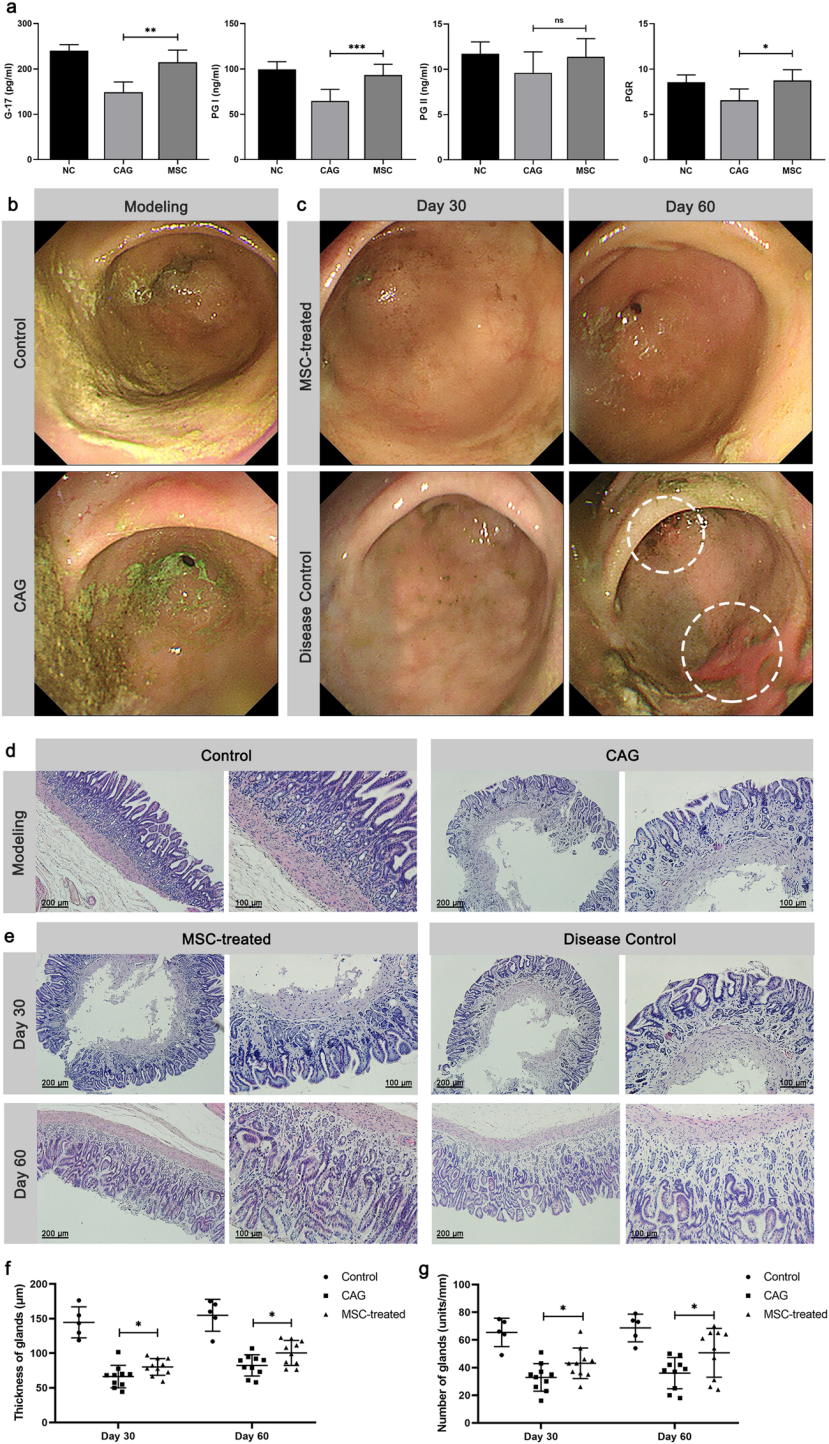
Therapeutic effects of MSCs submucosal injection on rabbit CAG. (**a**) Effect of MSCs on serum levels of G-17, PG I, PG II, and PGR. (**b**) Changes in the gastric mucosa after modeling. (**c**) Changes in the gastric mucosa following injection of MSCs or NS, the circle shown the mucosal ulcer. (**d**) Histopathological changes after modeling (H&E stain, × 100, × 200). (**e**) Effects of MSCs on gastric mucosal histopathological changes (H&E stain, × 100, × 200). (**f**) The thickness of gastric mucosa. (**g**) The gland number of gastric mucosa. Data were expressed as mean ± SD. *P < 0.05, **P < 0.01, ***P < 0.001 versus the CAG group (n = 5), ns means no significant difference. NC: negative control group; CAG: chronic atrophic gastritis group; MSC: MSC-treated group.

Activities of several specific markers in serum including G-17, PG I, and PG II were measured to determine whether there was atrophy of gastric mucosa. The results, presented in Fig. 2a, demonstrate a significant decrease in the levels of G-17 and PG I in the model group compared to the control group (*P* < 0.01). Although there was no statistically significant difference in PG II, the ratio of PG I and PG II (PGR) was decreased in the CAG group. In contrast, the serum levels of G-17, PG I, and PGR significantly increased in the MSC-treated group. Endoscopy examination revealed that the CAG mucosa was covered with more chyme, with mucosal edema, exposed blood vessels, and bile reflux (Fig. 2b). After 30 days of MSCs treatment, these mucosal changes reversed (Fig. 2c). Conversely, the disease control group showed multiple ulcerations, mucosal edema, and some hemorrhagic walls, which may be attributable to the poor mucosal status and repeated injections. Furthermore, a histopathological examination of the stomach was performed to directly demonstrate the therapeutic effects of MSCs against CAG (Fig. 2d–g). The glandular structure of the control group was complete and arranged as a whole, displaying a normal morphology of the gastric mucosa without atrophy. In the CAG group, the gastric mucosal epithelial cells were severely atrophied and necrotic (Fig. 2d), with a thin gastric mucosa and a significantly reduced number of glands, confirming the successful establishment of the CAG model. Pathologic changes were quantitatively assessed using thickness and numbers of glands, as illustrated in Fig. 2f and g. At 30 days after the initial treatment, the MSC-treated group exhibited significantly higher thickness of glands (80.21 ± 12.09 μm) and numbers of glands (42.1 ± 11.06 units/mm) in the gastric mucosa compared to the disease control group (thickness of glands: 66.19 ± 16.24 μm, numbers of glands: 32.9 ± 9.97 units/mm; *P* < 0.05). Following 60 days of MSCs treatment, a more pronounced recovery in both thickness of glands (100.43 ± 18.03 μm) and numbers of glands (50.7 ± 17.64 units/mm) was observed in comparison to the control group (thickness of glands: 62.36 ± 15.36 μm, numbers of glands: 36 ± 11.31 units/mm; *P* < 0.05). These results indicate that MSCs treatment has a reparative effect on gastric mucosal atrophy.

### Effect of MSCs on cell proliferation and migration of GES-1 cells

**Figure 3:**
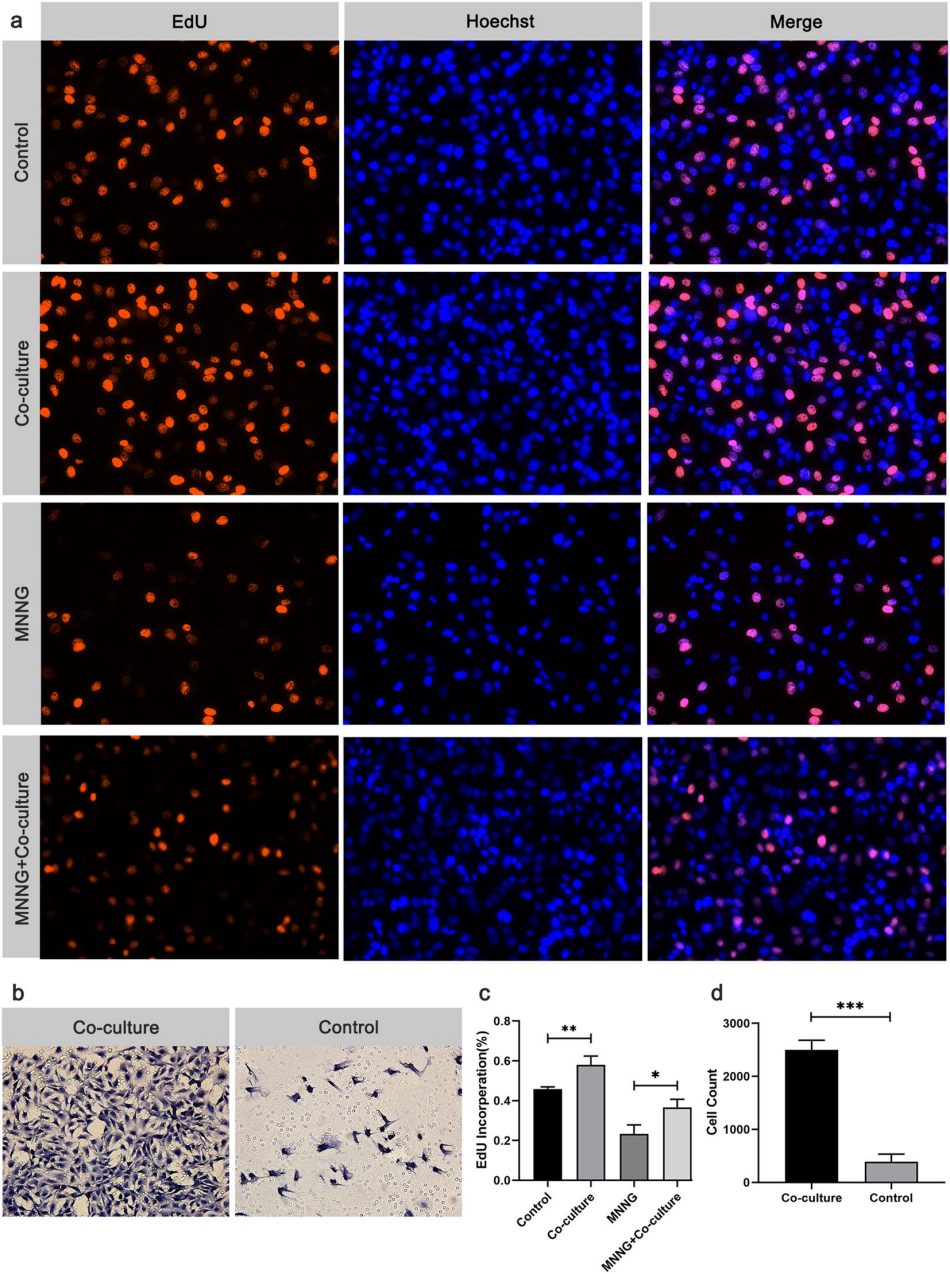
Effects of MSCs on the proliferation and migration of gastric epithelial cells. (**a**) GES-1 proliferation determined by EdU assay (× 400); Nucleus and DNA (living cells) were reflected by the staining fluorescence intensity of Hoechst 33,342 and EdU, respectively. (**b**) GES-1 migration determined by Transwell test (× 400). (**c**) Quantitative analysis for EdU-positive ratio. (**d**) Quantitative analysis of migration rate. *P < 0.05, **P < 0.01, ***P < 0.001.

To assess the influence of MSCs on the proliferation and migration of gastric mucosal epithelial cells, GES-1 and MSCs were plated in the upper and lower chamber respectively. After 24 h of co-culture, the EdU was performed. As shown in Fig. 3a,c, a higher percentage of EdU-positive proliferating GES-1 cells in co-culture group compared to the control group. Our results showed a significant increase in the percentage of EdU-positive proliferating GES-1 cells in the co-culture group compared to the control group (Fig. 3a,c). Moreover, co-culture with MSCs also enhanced cell proliferation after MNNG-induced injury. Next, we assessed the effect of MSCs on the migration ability of GES-1 cells using transwell chambers with a pore size of 3 μm. Our results demonstrated that GES-1 cells co-cultured with MSCs exhibited significantly enhanced migration capacity compared to the control group (Fig. 3b,d).

### The mechanisms of MSCs prevented GES-1 from MNNG-induced injury

**Figure 4:**
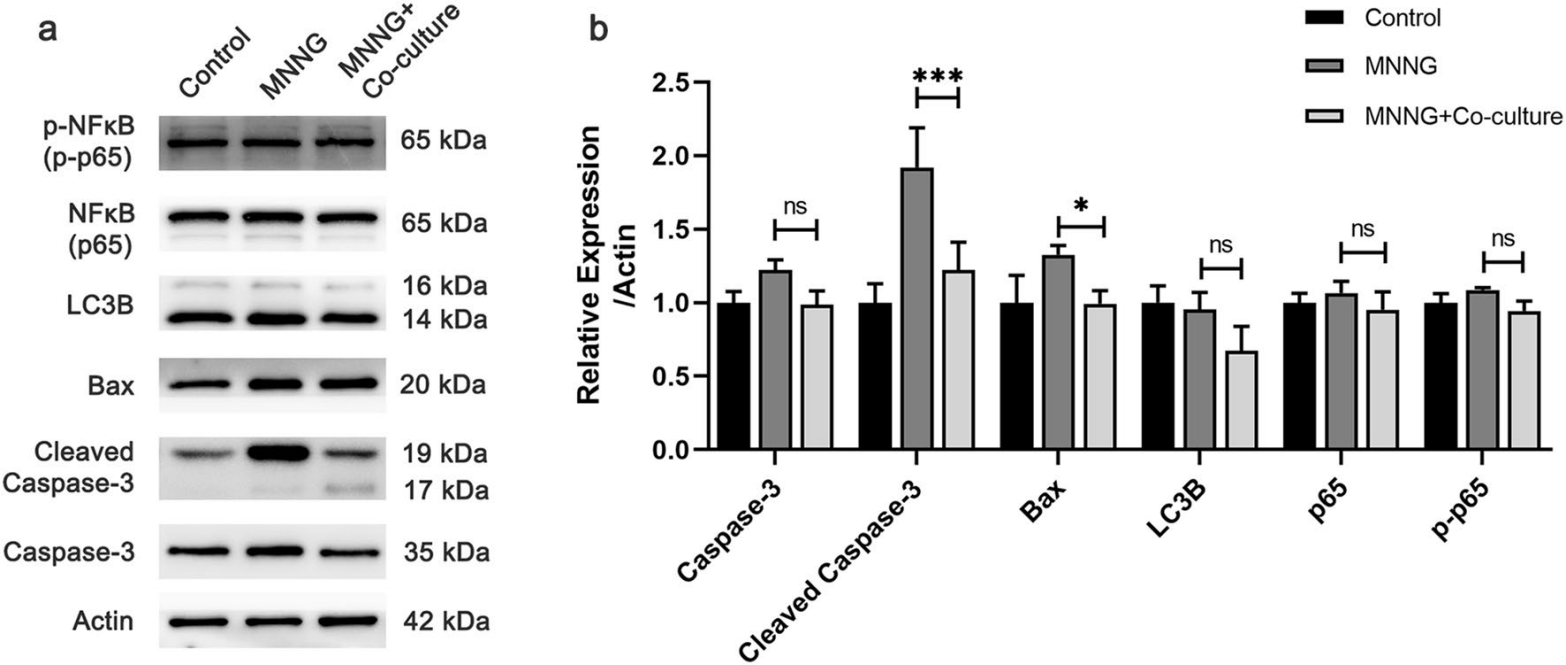
Effects of MSCs on apoptosis, autophagy, and inflammation. (**a**) Western blot for caspase-3, cleaved caspase-3, Bax, LC3B, p65, and p-p65. (**b**) Quantitative analysis of protein expression.

One of the hallmark features of atrophic mucosa is the loss of glandular regenerative capacity, which can result from increased apoptosis, dysregulated cell cycle progression, and reduced cell survival. To explore the mechanism underlying the response of GES-1 cells to co-culture with MSCs, we examined the expression of several key indicators involved in apoptosis, autophagy, and inflammation. Figure 4 shows that MNNG treatment upregulated the relative expression levels of cleaved caspase-3 and Bax, whereas co-culture with MSCs mitigated this increase (*P* < 0.01). However, we noted that there were no significant changes in the autophagy marker LC3B or the inflammatory marker phosphor-p65 among the three groups (Supplementary file).

## Discussion

Gastric cancer is a significant cause of global morbidity and mortality, and its development is closely linked to various pre-neoplastic conditions^1^. Among them, CAG is the most common and exceedingly precancerous diseases that can result from H. pylori infection, bile reflux, or autoimmune reactions. Recent follow-up studies have demonstrated that the annual incidence of gastric cancer in patients with CAG ranges from 0.1 to 0.3%^12,13^. Therefore, CAG is a key point in the prevention of gastric cancer, and prompt treatment can significantly reduce the incidence of cancer. H. pylori infection is recognized as the most critical factor in the development of CAG. Previous studies have shown that H. pylori eradication can reverse atrophy and intestinal metaplasia to some extent^4,14^. However, the reversal rate following eradication is influenced by the severity and extent of atrophy^15^. In recent years, traditional Chinese medicine (TCM) has gained increasing attention as a potential treatment for CAG^16–18^. However, the precise molecular mechanisms underlying the therapeutic effects of TCM remain unclear, and large-scale clinical studies are required to facilitate its application in CAG clinical treatment.

MSCs have garnered considerable attention in the field of regenerative medicine due to their remarkable regenerative potential, immunomodulatory properties, and ability to differentiate into multiple cell lineages^9^. Several studies have highlighted the rejuvenating and restorative effects of MSCs against various gastric damaging conditions, including radiation-induced gastrointestinal injuries^19^, gastrointestinal damages after ischemia–reperfusion^20^, and NSAIDs-induced gastrointestinal damages^21^. Notably, a recent study by Park et al. demonstrated the significant therapeutic potential of placenta-derived MSCs in H. pylori-induced CAG, with superior efficacy compared to UC-MSCs and AD-MSCs^22^. However, current studies are mostly based on intravenous administration of MSCs. According to tracer studies, system refusion for MSCs results in poor homing and compromises desirable effects on target organs, especially stomach^23^. Also, circulation and immune elimination may reduce cell survival before cells become functional^24^. These limited the therapeutic benefits of MSCs therapy in gastric diseases. To overcome these limitations, we aimed to improve the homing efficiency of MSCs in the stomach by endoscopic submucosal injection, taking advantage of the widespread use of endoscopy in gastroenterology as a diagnostic and therapeutic tool.

Here, we introduced a rabbit model of MNNG-related atrophic gastritis, which has been widely used to simulate the transformation of normal gastric mucosa cells into malignant cells. After 12 weeks of modeling, we observed significant changes in the gastric mucosa, including edema, thinning, paleness, erosive mucosa, glandular atrophy, and infiltration of inflammatory cells, mostly lymphocytes. Local injection of MSCs significantly alleviated these changes, while saline injection worsened the gross appearance and histopathology of the gastric mucosa compared to the control group. These results are in line with previous studies on the therapeutic effects of MSCs in CAG treatment and provide a promising approach for improving the homing efficiency of MSCs in gastric diseases.

In the context of MSCs therapy, the mechanisms underlying tissue repair remain a subject of debate, with controversy surrounding whether the therapeutic effect is mediated primarily through cell differentiation or via a paracrine effect with the release of trophic factors. In our study, we investigated the effects of MSCs on gastric mucosal cells and found that MSCs promoted cell proliferation and migration while down-regulating cell apoptosis, consistent with the notion that MSCs primarily facilitate tissue repair through paracrine mechanisms. Specifically, transplanted MSCs secrete cytokines that create a local microenvironment conducive to tissue healing, influencing stem/progenitor cell survival, proliferation, and differentiation, and protecting histocytes from apoptosis and cell death.

Surprisingly, we did not observe significant anti-inflammatory effects of MSCs in our results, as evidenced by unchanged levels of the p65 or phosphor-p65 protein markers. Additionally, we found no significant differences in the autophagy marker LC3B, despite the importance of autophagy in maintaining mucosal homeostasis and immunity. Prior studies have suggested that changes in autophagy are implicated in the pathogenesis of CAG^25^, but the therapeutic effects of autophagy in CAG remain controversial. It is important to note that our in vitro model may have limitations in reflecting the actual effects of MSCs in vivo, given the lack of simulation of the gastric mucosal and internal physiological environment.

One limitation of this study is that the precise therapeutic mechanism of MSCs and their interaction with tissue-resident cells were not fully elucidated. Therefore, before translating these findings to clinical practice, it is essential to have a thorough understanding of the mechanisms involved in the therapeutic effect. Additionally, while our study demonstrates the potential of endoscopic submucosal injection of MSCs in reversing CAG, it is important to acknowledge the possible risks associated with multiple injections, such as hyperplasia and rejection. The optimal treatment course, dosing, and follow-up period require further investigation. Furthermore, it’s worth noting that our pathological assessment mainly focused on glandule thickness and numbers as quantitative measures. Additional quantitative parameters, such as the extent of inflammatory cell infiltration, counts of chief cells and parietal cells, or the degree of intestinal metaplasia, were not included. These additional measures could have provided a more comprehensive evaluation of gastric mucosal pathology. Future research endeavors may benefit from incorporating a broader range of quantitative parameters for a more detailed and specific characterization of these lesion changes.

In summary, our study provides evidence that endoscopic submucosal injection of MSCs may be a promising therapeutic strategy for CAG by promoting cellular proliferation and suppressing apoptosis. Our findings also suggest that the use of MSCs could potentially inhibit the progression of pre-neoplastic lesions. Future studies involving larger animal models and clinical trials are warranted to further investigate the clinical potential of this approach.

### Supplementary Information

Supplementary Information 1.

## Data Availability

All data associated with this study are present in the paper.

## Abbreviations

CAG: Chronic atrophic gastritis
IM: Intestinal metaplasia
MSCs: Mesenchymal stem cells
