# Supplementary figures and images for: Therapeutic effects and potential mechanisms of endoscopic submucosal injection of mesenchymal stem cells on chronic atrophic gastritis
Source: Sci Rep. 2023 Nov 25;13:20745. doi: 10.1038/s41598-023-48088-3 (PMC10676420; doi:10.1038/s41598-023-48088-3)

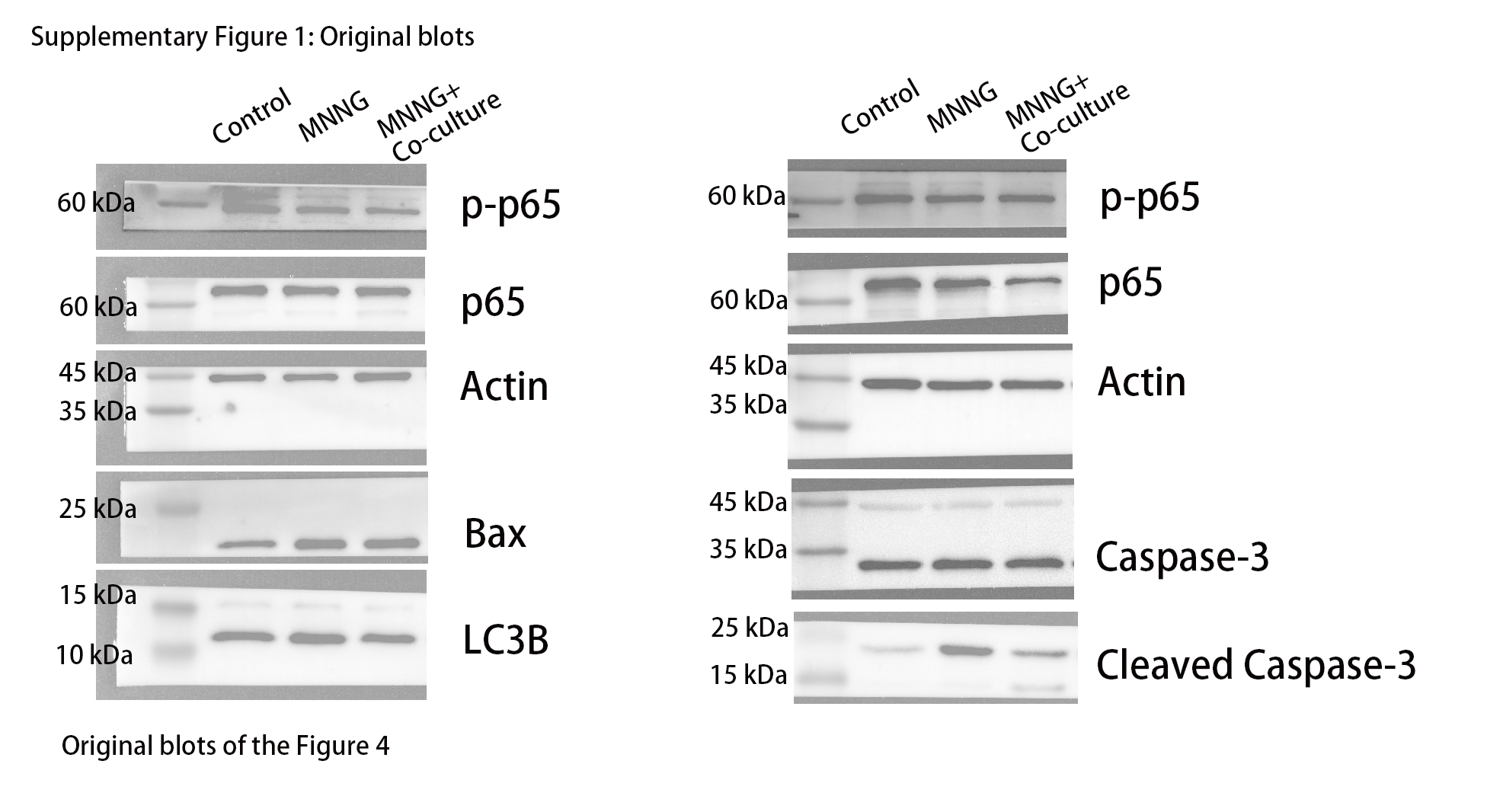

Supplement: Supplementary file 1 — Supplementary Information 1. [file 41598_2023_48088_MOESM1_ESM.tif]
